# Retrospective evaluation of Facial nerve monitoring to prevent nerve damage during robotic drilling in the largest series of patients undergoing the HEARO-procedure

**DOI:** 10.1371/journal.pone.0326614

**Published:** 2025-06-26

**Authors:** Jaouad Abari, Marco Matulic, Pablo Galeazzi, Masoud Zoka Assadi, Paul Van de Heyning, Vedat Topsakal

**Affiliations:** 1 Department of Otorhinolaryngology, Head and Neck Surgery, University Hospital UZ Brussel, Vrije Universiteit Brussel, Brussels, Belgium; 2 CASCINATION AG, Bern, Switzerland; 3 MED-EL Medical Electronics, Innsbruck, Austria; 4 Department of Otorhinolaryngology, Head and Neck Surgery, Antwerp University Hospital, University of Antwerp, Antwerp, Belgium; 5 Vrije Universiteit Brussel, Brussels Health Campus, Brussels, Belgium; 6 Department of Otorhinolaryngology, Head and neck Surgery, Clinique Universitaire Saint Luc, Brussels, Belgium; 7 Institute of Neuroscience, Université catholique de Louvain, Brussels, Belgium; Sapienza University of Rome: Universita degli Studi di Roma La Sapienza, ITALY

## Abstract

**Introduction:**

Robot-assisted cochlear implantation surgery (RACIS) involves the drilling of a keyhole access to the inner ear for cochlear implant placement to treat patients with severe-to-profound sensorineural hearing loss. RACIS with the HEARO-procedure does not require the drilling of a mastoidectomy and posterior tympanotomy to pass through the facial recess. Instead, it directly drills through it guarding a safe distance from both the facial nerve and chorda tympani. Cochlear implantation surgery involves a well described risk for facial nerve injury when passing through the facial recess. Neuromonitoring as a safety protocol gained great importance in conventional CI surgery and is proving its benefits in RACIS. RACIS in the HEARO-procedure involves a customized facial nerve monitoring (FNM) device that was designed and tested in an animal model study. Here, this device was retrospectively assessed in the largest series of patients undergoing the HEARO-procedure.

**Materials and methods:**

The safety protocol in the HEARO-procedure involves FNM and intra-operative cone-beam CT (CBCT) imaging with a 0.1 mm spatial resolution. The customized FNM device was employed, using both active mono- and bipolar stimulation to estimate the distance to the facial nerve in RACIS. Linear regression was used to determine if the minimum stimulation thresholds (FNM) could significantly predict the intra-operative distance (CBCT) between the drilled trajectory and the facial nerve. Logistic regression was used to calculate if FNM can distinguish distances smaller and greater than 0.4 mm to the facial nerve.

**Results:**

The minimum stimulation thresholds significantly predicted the distances between the drilling trajectory and the facial nerve for both the monopolar (p = 0.001) and bipolar 3 (p = 0.008) stimulation configuration. Both the monopolar (β = -0.189, S.E. = 0.063, p = 0.003) and bipolar 3 (β = -0.187, S.E. = 0.080, p = 0.019) stimulation configuration are negative and significant predictors of the probability of the distance being smaller than 0.4 mm.

**Conclusion:**

FNM will alert the surgeon when the drilling trajectory comes closer than 0.4 mm to the facial nerve in RACIS. A linear relationship was observed between the minimum stimulation thresholds and the intra-operative distance towards the facial nerve.

## Introduction

The World Health Organisation estimates that around 5% of the worlds population has some kind of a disabling hearing loss [1]. Although well-established reimbursement programs for hearing aids and cochlear implants are available in most western countries, the uptake rate remains low [2]. On the other hand, there are more than a million cochlear implant users today [3]. Cochlear implantation (CI) is widely accepted as the most effective treatment for patients with severe to profound sensorineural hearing loss [4]. Its surgical placement is low risk involving an enlarged mastoidectomy followed by a posterior tympanotomy. This so-called facial recess approach is considered the gold standard technique [5,6]. Performed by an experienced otologist, the risk of complications in conventional cochlear implantation surgery is low, with a facial nerve injury prevalence of less than 1% [7,8]. This is probably due to the rather stable and not too much alternating anatomical position of the facial nerve, allowing a trained otologist to respect it during the drilling of the mastoid. A free running facial nerve monitor is used during the conventional procedure which can give an alarm when the surgeon accidentally drills on the facial nerve.

Advancements in image guided surgery allowed for the development of robot-assisted cochlear implantation surgery (RACIS) [9]. New sets of tools were engineered to make the HEARO®-procedure (CAScination, Bern, Switzerland) a reality. OTOPLAN® (CAScination, Bern, Switzerland), initially a 3D planning software programme used to calculate the surgical trajectory is now even commercially available as a stand-alone product for otologists as a bedside image handling tool [10]. The HEARO-procedure allows access to the inner ear by robotic drilling a direct keyhole trajectory through the mastoid and facial recess. Electrode array insertion is performed through this keyhole trajectory either manually or robotically with another tool [11].

High-resolution imaging empowered with highly accurate navigation systems enable the robotic drilling of a 1.8 mm keyhole trajectory through the mastoid and facial recess for middle ear access [12–14]. Hereafter, inner ear access is achieved after the milling of a 1.0 mm keyhole trajectory through the bony overhang of the round window [15]. Electrode array insertion can thus be achieved through this minimal invasive access into the inner ear. The accuracy of the robotic drilling arm was calibrated to be 0.15 ± 0.08 mm when verified in pre-clinical trials on human cadavers [16,17]. The size of the facial recess at the level of the round window is described as 2.65 ± 0.63 mm [18]. During the HEARO® procedure, drilling through the facial recess is considered the most critical step. A custom-made drill with a diameter of 1.8 mm must pass at a safe distance of at least 0.4 mm and 0.3 mm from both the facial nerve and the chorda tympani respectively. These safety margins are described as being three standard deviations larger than the accuracy of the robotic system [13,15]. Drilling through the facial recess is performed in 0.5 mm intervals.

The importance of neuromonitoring as a safety protocol is important in both conventional and robotic cochlear implantation surgery. For the HEARO-procedure, a customized facial nerve monitoring (FNM) device was used, employing both active mono- and bipolar stimulation. In a prospective study on sheep, it was demonstrated that FNM was able to prevent structural damage to the facial nerve. In 4 out of 7 sheep, FNM was able to correctly estimate facial nerve proximity for distances less than 0.1 mm. A prediction of facial nerve proximity can thus be made depending on the stimulation threshold used. Smaller stimulation thresholds correlated with smaller distance ranges, and vice versa [19].

Here, we studied the efficacy and reliability of the customized FNM device during the HEARO procedure in the largest series of patients undergoing RACIS to treat their severe to profound hearing loss.

## Materials and methods

### Study design and ethical considerations

This study is set up as a retrospective case-control study. The approval of the UZ Brussel ethics committee was granted with number BUN: 1432022000276. The ethics committee waived the requirement for informed consent. The data for this study was accessed on 4 September 2023. The authors did not have access to information that could identify individual participants during or after data collection due to data anonymization.

The aim is to assess the performance of the FNM device in our database of patients who have underwent RACIS with the HEARO-procedure and to determine the correlation between the stimulation thresholds at which a response was measured and the distance to the facial nerve measured using intra-operative imaging.

### Intra-operative cone-beam CT

**Fig 1:**
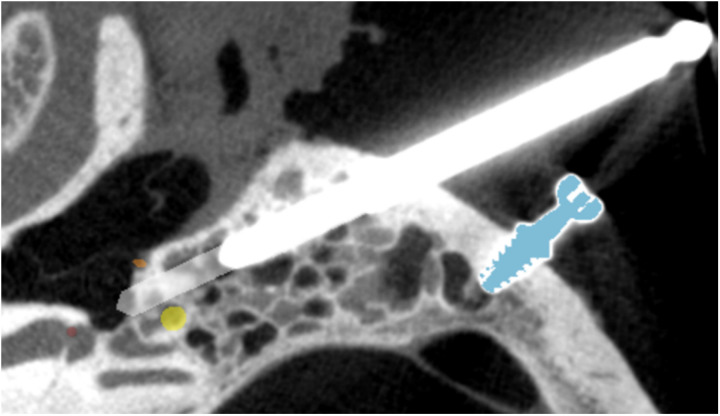
Intra-operative cone-beam CT of titanium rod placed inside the drilling trajectory and calculation of the facial nerve (yellow) distance using a dedicated 3D planning software programme. Fiducials (blue) are used for patient to image registration. The chorda tympani (orange) and the target/cochlea (red) are also visualized.

Before robotically drilling through the facial recess, imaging is performed in all included cases with a mobile cone-beam CT with a 0.1 mm spatial resolution (XCAT XL, Xoran Ltd, Ann Arbor, Michigan, USA). A titanium rod is placed in the partially drilled tunnel, 3 mm before reaching the facial recess, to enhance the contrast of the keyhole trajectory (Fig 1). The intra-operative distance between the drilled trajectory and the facial nerve for each RACIS case is measured using a dedicated 3D planning software programme [13,15].

### Facial nerve monitoring procedure

**Fig 2:**
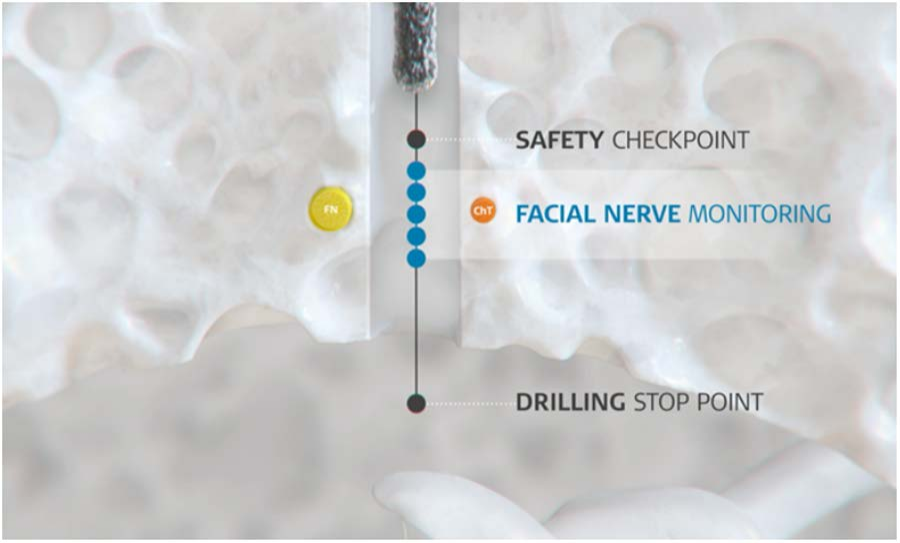
FNM is performed after each 0.5 mm drilling interval (blue point) when drilling through the facial recess. The facial nerve is indicated in yellow and the chorda tympani in orange.

**Fig 3:**
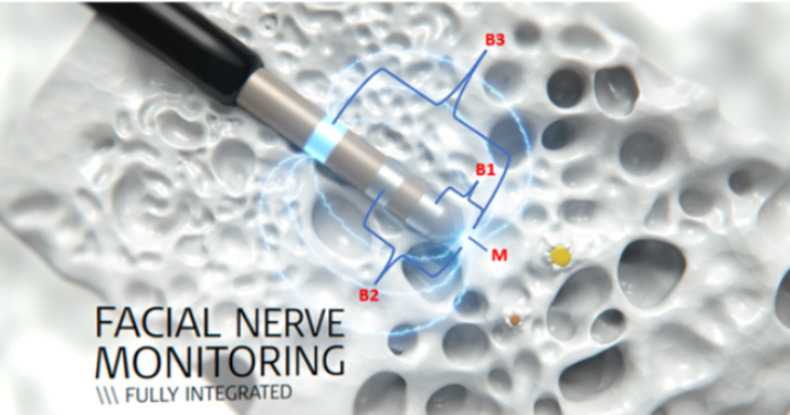
The FNM probe features four different stimulation configurations: monopolar (M), bipolar 1 (B1), bipolar 2 (B2) and bipolar 3 (B3).

A pair of subdermal electromyography needles are inserted into the orbicularis oculi muscle and a pair into the orbicularis oris muscle. The correct placement of these needle electrodes is checked by directly stimulating the facial nerve. Drilling through the facial recess is conducted in small 0.5 mm intervals starting 3 mm in front of the estimated depth of the facial nerve. After each 0.5 mm interval, active facial nerve stimulation is performed using the FNM probe that is inserted in the drilled trajectory (Fig 2). The FNM probe features four different stimulation configurations: the monopolar (M), bipolar 1 (B1), bipolar 2 (B2) and bipolar 3 (B3) stimulation (Fig 3). Each configuration will produce a stimulation threshold ranging from 0.2 to 2.0 mA. The compound muscle action potential (CMAP) can then be measured from both facial muscles in millivolt (mV). Excluding artefacts, minimum stimulation thresholds that elicit a CMAP response above 0.1 mV are recorded as an event. A CMAP response of less than 0.1 mV at a stimulation threshold of 2 mA is not considered a significant event and indicates that the measurement site is not in close proximity to the facial nerve. A total of five measurements are performed for each stimulation configuration.

Conventional monopolar neuromonitoring alone cannot accurately estimate the proximity to the facial nerve at distances less than 0.1 mm [20]. While monopolar stimulation provides high sensitivity, it lacks specificity. By using 3 electrodes for bipolar stimulation at lower thresholds, more specific estimations of distances smaller than 0.5 mm can be achieved [21].

### Data-analysis

All cases were performed by the same surgeon (VT) with the HEARO®-procedure (CAScination, Bern, Switzerland) as previously published [15]. Cases that had to be converted to conventional cochlear implantation surgery before drilling through the facial recess and where there is thus no FNM data to analyse had to be excluded from the analysis.

The following data were analysed for the monopolar, bipolar 1, bipolar 2 and bipolar 3 configuration:

- The intra-operative distance (mm) between the drilled trajectory and the facial nerve for each RACIS case measured during intra-operative cone-beam CT imaging.
- The CMAP responses (mV) measured at both the orbicularis oculi and orbicularis oris muscles.
- The minimum stimulation threshold (mA) that elicited an event: a CMAP response above 0.1 mV at each interval.

A total of 33 patients underwent RACIS. Three cases were excluded from this study. Two were excluded due to a software failure disabling the intra-operative safety assessments. The third case was excluded because of intra-operative imaging not being able to predict a safe distance towards the facial nerve. All excluded cases were successfully implanted through conventional cochlear implantation surgery. The data consisted of 30 cases or 600 measurements. Only 192 events or minimum stimulation thresholds (mA) that elicited a CMAP response above 0.1 mV were analysed.

### Statistical considerations

All events of each drilling interval were analysed together using linear regression and logistic regression analysis. Linear regression was used to determine if the minimum stimulation thresholds that elicited a CMAP response above 0.1 mV significantly predicted the intra-operative distance between the drilled trajectory and the facial nerve. Logistic regression was used to calculate if facial nerve monitoring can distinguish distances smaller and greater than 0.4 mm.

## Results

In none of the patients undergoing the HEARO-procedure was there a case of structural or functional damage to the facial nerve. In two cases, FNM alarmed the surgeon for an unsafe trajectory reaching closer than 0.4 mm to the facial nerve which is considered unsafe according to the current safety mitigation protocol. Intra-operative imaging and 3D planning in the first case confirmed the distance towards the facial nerve to be 0.222 mm. Robotic drilling through the facial recess was continued but was aborted after the third facial nerve stimulation interval. At this point, each respective stimulation configuration demonstrated a minimum stimulation threshold of 0.2 mA.

**Fig 4:**
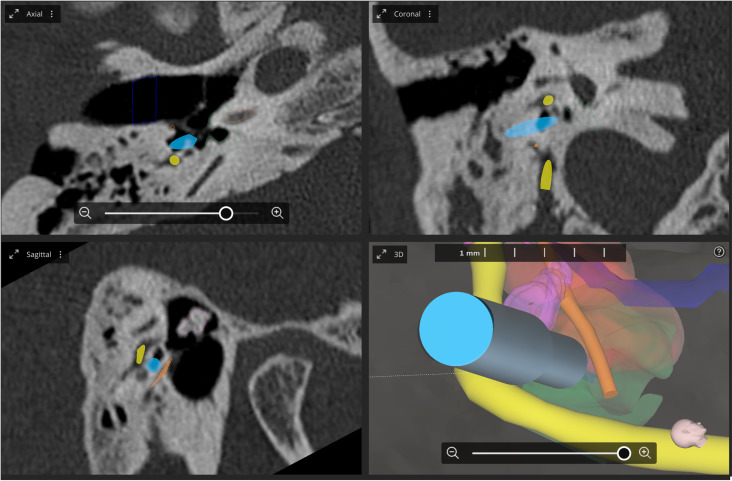
Intra-operative cone-beam CT and 3D reconstruction of incorrectly partially segmented facial nerve (yellow). The drilling trajectory (light blue) was in reality closer to the facial nerve than initially calculated.

In the second case, intra-operative imaging and 3D planning predicted the distance towards the facial nerve to be 0.415 mm. Robotic drilling through the facial recess was continued but also aborted after the third facial nerve stimulation interval. At this point, facial nerve monitoring could not warrant a safe distance towards the facial nerve. The monopolar, bipolar 1 and 2 stimulation configuration demonstrated a minimum stimulation threshold of 1.25 mA, 0.3 mA and 0.75 mA respectively. A post-operative analysis was performed because of the discrepancy between the FNM responses and the measured distance with cone-beam CT imaging from the facial nerve. This analysis demonstrated the facial nerve to have been incorrectly partially segmented which resulted in a miscalculated safe trajectory estimation. A section of the facial nerve was thus closer to the drilling trajectory than measured (Fig 4). The actual intra-operative distance to the facial nerve was 0.290 mm. In both cases the HEARO-procedure was converted to conventional surgery. Both patients were successfully implanted without any complications.

### Monopolar configuration

**Fig 5:**
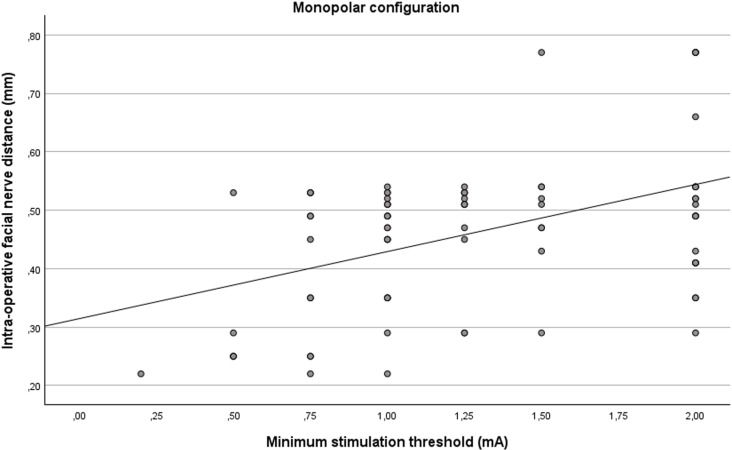
Linear relationship between intra-operative distance from the facial nerve and the minimum stimulation thresholds for the monopolar configuration.

Simple linear regression analysis was statistically significant (R^2^ = 0.202, F(1, 80) = 20.24, p = 0.001) for the monopolar configuration (Fig 5). The minimum stimulation thresholds significantly predicted the distances from the facial nerve (β = 0.449, p = 0.001).

The minimum stimulation thresholds in the monopolar configuration are a negative and significant predictor of the probability of the distance being smaller than 0.4 mm (β = −0.189, S.E. = 0.063, p = 0.003).

The odds ratio for the minimum stimulation thresholds is 0.828 (C.I. = [0.732, 0.937]) meaning that the odd of the distance being smaller than 0.4 mm decreases by 17,2% with every 0.1 mA increase in the minimum stimulation threshold.

### Bipolar 1 configuration

Simple linear regression was not statistically significant (R^2^ = 0.164, F(1, 17) = 3.34, p = 0.085) for the bipolar 1 configuration. The minimum stimulation thresholds did not significantly predict the distances from the facial nerve (β = 0.405, p = 0.085).

The minimum stimulation thresholds in the bipolar 1 configuration are a negative but non-significant predictor of the probability of the distance being smaller than 0.4 mm (β = −0.168, S.E. = 0.102, p = 0.099). The odds ratio for the minimum stimulation thresholds is 0.846 (C.I. = [0.693, 1.032]) meaning that the odd of the distance being smaller than 0.4 mm decreases by 15,4% with every 0.1 mA increase of the minimum stimulation threshold.

### Bipolar 2 configuration

Simple linear regression was not statistically significant (R^2^ = 0.099, F(1, 36) = 3.95, p = 0.055) for the bipolar 2 configuration. The minimum stimulation thresholds did not significantly predict the distances from the facial nerve (β = 0.214, p = 0.055).

The minimum stimulation thresholds in the bipolar 2 configuration are a negative but non-significant predictor of the probability of the distance being smaller than 0.4 mm (β = −0.129, S.E. = 0.074, p = 0.083). The odds ratio for the minimum stimulation thresholds is 0.879 (C.I. = [0.760, 1.017]) meaning that the odd of the distance being smaller than 0.4 mm decreases by 12.1% with every 0.1 mA increase of the minimum stimulation threshold.

### Bipolar 3 configuration

**Fig 6:**
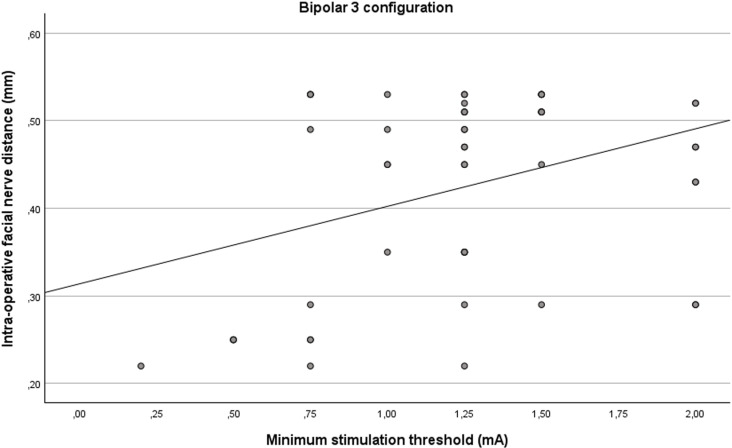
Linear relationship between intra-operative distance from the facial nerve and the minimum stimulation thresholds for the bipolar 3 configuration.

Simple linear regression was statistically significant (R^2^ = 0.130, F(1, 51) = 7.65, p = 0.008) for the bipolar 3 configuration (Fig 6). The minimum stimulation thresholds significantly predicted the distances from the facial nerve (β = 0.361, p = 0.008).

The minimum stimulation thresholds in the bipolar 3 configuration are a negative and significant predictor of the probability of the distance being smaller than 0.4 mm (β = −0.187, S.E. = 0.080, p = 0.019). The odds ratio for the minimum stimulation thresholds is 0.829 (C.I. = [0.710, 0.970]) meaning that the odd of the distance being smaller than 0.4 mm decreases by 17,1% with every 0.1 mA increase of the minimum stimulation threshold.

## Discussion

Here we present the clinical assessment of multipolar FNM for estimating facial nerve proximity during RACIS in the largest series of patients. Robotizing surgical tasks is gaining more and more popularity. As described by Fosch-Villaronga et al., surgical automation is categorized into 5 levels [22]. Most surgical robots in use today operate at autonomy level 2. In this setup, there is a master-slave connection where the surgeon (master) remotely controls a robotic tool (slave). With a low level of robotic autonomy, the need for a rigorous safety protocol is less crucial [23]. In the HEARO procedure, as described by Topsakal et al. [15], the autonomy level is 3, where the surgeon still manually segments the patients anatomy to determine a surgical trajectory towards the inner ear but the drilling is performed by a robotic arm. The safety mitigation protocol in robotic procedures need perhaps to be more rigorous because of potential human errors during surgical trajectory segmentation. In this sense a secondary and independent safety measure is given by the FNM.

Significant results were observed for the monopolar and bipolar 3 stimulation configurations in our series of cases. A linear relationship was established between the minimum stimulation thresholds that elicited a CMAP response above 0.1 mV and the intra-operative distance relative to the facial nerve. As expected, a response following increasing stimulation thresholds correlated with a greater intra-operative distance, and vice versa. Both the monopolar and bipolar 3 stimulation configurations were able to significantly distinguish distances greater and smaller than 0.4 mm.

On the scatterplot, some variability is observed in the distances relative to the facial nerve per stimulation threshold. The initial pre-clinical FNM trial was calibrated on sheep [19]. Although the anatomy of a sheep’s mastoid is comparable to that of a human [24]. We suspect the presence of air cells (where there are no air cells in a sheep’s mastoid) and the differences in bone quality to have an effect on how the stimulation threshold is transmitted to the facial nerve and on the measured responses. The subtle variations in the anatomy determine the different media that the electrical current must traverse. This could explain this observed variability.

Most measurements represent distances greater than 0.4 mm. Although the results are statistically significant, FNM performance could only be evaluated on a limited set of critical distances. This is to be expected because preoperatively, a minimum safety margin of at least 0.4 mm in relation to the facial nerve is considered. The prospective study in sheep was able to include more critical distances, but for obvious reasons this is not possible in humans. However, it can be stated that in the two cases where there was a critical distance relative to the facial nerve, FNM was able to alert the surgeon in time. Because of this, the surgeon decided to convert these HEARO-procedures to conventional surgery without any complications. Regardless of how performant or accurate the navigation system is, anecdotal accidents and injuries have been observed when passing the facial nerve using only a navigation system. It seems indispensable to have two diverse systems for risk mitigation that are independent of each other.

The aim of the HEARO-procedure is not to replace the surgeon but to be used as a surgical instrument that increases the quality and reliability of the surgery standardising outcomes for patients. Based on the patients’ individual anatomy and cochlear parameters, a personalised trajectory to the inner ear is calculated [25]. The angle of insertion can be warranted better than any surgeon can perform or estimate using the anatomical landmarks, simply because it is not possible to look beyond the level of the round window with a microscope. The required accuracy and sensitivity during cochlear implantation is already at the limits of human dexterity. With electrode arrays becoming smaller in the future, the need for robotic accuracy and reliability will become necessary. Robotic cochlear implantation surgery would take less than 10 minutes for inner ear access however the current safety protocols such as FNM and intra-operative imaging require most of the time. This study evaluating FNM could help reduce RACIS time. Perhaps RACIS can become an outpatient procedure under local anaesthesia.

RACIS should not only respect the anatomical course of the facial nerve but should also not cause any thermal injury during drilling. Drilling through temporal bone close to the facial nerve can lead to a harmful temperature rise [26]. Facial nerve palsy due to the heat generated during drilling has been described in the past, in a case of template based cochlear implantation [27]. Therefore, a custom-made helical step drill with an optimized geometry was designed to minimize the heat buildup during drilling. Furthermore, drilling is performed in 0.5 mm intervals with additional flushing of the drilled trajectory to prevent chip-build up and thus to further minimize harmful temperatures [28].

With the cone-beam CT that has a spatial resolution of 0.1 mm, a safe distance from the facial nerve can still be planned with great accuracy. However, when compared to the prospective study on sheep that used a µCT imaging device with a spatial resolution of 0.018 mm, the resolution is 5 times lower. In terms of the safety of the procedure, this has no implications. The drilling accuracy has been verified to be 0.1 ± 0.05 mm excluding image-related errors and 0.15 ± 0.08 mm on human cadavers (6). Besides the difference in spatial resolution, µCT imaging is also not feasible in an intra-operative setting.

The most recent version of the planning software can automatically segment the crucial anatomy of the inner ear and suggest a drilling trajectory. Here, the surgeon needs to approve the suggested drilling trajectory and supervise its execution reaching towards a level 4 of robotic autonomy. An automated segmentation of the patients anatomy and surgical trajectory to the level of a voxel eliminates any potential human error or inaccuracy. Higher autonomy levels require separate rigorous safety mitigation protocols. Current FNM and perhaps newer electrophysiological monitoring devices will therefore, continue to play a crucial role in RACIS.

## Conclusions

A linear relationship was observed between the minimum stimulation thresholds and the intra-operative distance towards the facial nerve in this study analysing RACIS. The customized FNM device can distinguish distances greater and smaller than 0.4 mm.

## References

[1] WHO. Deafness and hearing loss; 2021. https://www.who.int/news-room/fact-sheets/detail/deafness-and-hearing-loss.

[2] D’Haese P, Topsakal V, Greenham P. The World Report on Hearing, what does it mean for me and how can it improve access to hearing devices? Ear Nose Throat J. 2023. doi: 1455613231157937 10.1177/01455613231157937 36790318

[3] Zeng F-G. Celebrating the one millionth cochlear implant. JASA Express Lett. 2022;2(7):077201. doi: 10.1121/10.0012825 36154048

[4] Gaylor JM, Raman G, Chung M, Lee J, Rao M, Lau J, et al. Cochlear implantation in adults: a systematic review and meta-analysis. JAMA Otolaryngol Head Neck Surg. 2013;139(3):265–72. doi: 10.1001/jamaoto.2013.1744 23429927

[5] Clark GM, Pyman BC, Bailey QR. The surgery for multiple-electrode cochlear implantations. J Laryngol Otol. 1979;93(3):215–23. doi: 10.1017/s0022215100086977 429901

[6] Jansen C. Posterior tympanotomy: experiences and surgical details. Otolaryngol Clin North Am. 1972;5(1):79–96. doi: 10.1016/s0030-6665(20)33020-6 4551418

[7] Alzhrani F, Lenarz T, Teschner M. Facial palsy following cochlear implantation. Eur Arch Otorhinolaryngol. 2016;273(12):4199–207. doi: 10.1007/s00405-016-4124-0 27276989

[8] Thom JJ, Carlson ML, Olson MD, Neff BA, Beatty CW, Facer GW, et al. The prevalence and clinical course of facial nerve paresis following cochlear implant surgery. Laryngoscope. 2013;123(4):1000–4. doi: 10.1002/lary.23316 23382004

[9] Weber S, Gavaghan K, Wimmer W, Williamson T, Gerber N, Anso J, et al. Instrument flight to the inner ear. Sci Robot. 2017;2(4):eaal4916. doi: 10.1126/scirobotics.aal4916 30246168 PMC6150423

[10] Mertens G, Van Rompaey V, Van de Heyning P, Gorris E, Topsakal V. Prediction of the Cochlear Implant Electrode Insertion Depth: Clinical Applicability of two Analytical Cochlear Models. Sci Rep. 2020;10(1):3340. doi: 10.1038/s41598-020-58648-6 32094372 PMC7039896

[11] Abari J, Heuninck E, Topsakal V. Entirely robotic cochlear implant surgery. Am J Otolaryngol. 2024;45(5):104360. doi: 10.1016/j.amjoto.2024.104360 38754261

[12] Caversaccio M, Gavaghan K, Wimmer W, Williamson T, Ansò J, Mantokoudis G, et al. Robotic cochlear implantation: surgical procedure and first clinical experience. Acta Otolaryngol. 2017;137(4):447–54. doi: 10.1080/00016489.2017.1278573 28145157

[13] Caversaccio M, Wimmer W, Anso J, Mantokoudis G, Gerber N, Rathgeb C, et al. Robotic middle ear access for cochlear implantation: First in man. PLoS One. 2019;14(8):e0220543. doi: 10.1371/journal.pone.0220543 31374092 PMC6677292

[14] Tekin AM, Bleys RLAW, Matulic M, Assadi MZ, van de Heyning P, Bahşi İ, et al. Next-generation Robotics in Otology: The HEARO Procedure. J Craniofac Surg. 2025;36(1):138–45. doi: 10.1097/SCS.0000000000010887 39591381

[15] Topsakal V, Heuninck E, Matulic M, Tekin AM, Mertens G, Van Rompaey V. First study in men evaluating a surgical robotic tool providing autonomous inner ear access for cochlear implantation. Frontiers in Neurology. 2022;13. 10.3389/fneur.2022.804507 PMC8979022 35386404

[16] Wimmer W, Venail F, Williamson T, Akkari M, Gerber N, Weber S, et al. Semiautomatic cochleostomy target and insertion trajectory planning for minimally invasive cochlear implantation. Biomed Res Int. 2014;2014:596498. doi: 10.1155/2014/596498 25101289 PMC4101975

[17] Bell B, Gerber N, Williamson T, Gavaghan K, Wimmer W, Caversaccio M, et al. In vitro accuracy evaluation of image-guided robot system for direct cochlear access. Otol Neurotol. 2013;34(7):1284–90. doi: 10.1097/MAO.0b013e31829561b6 23921934

[18] Bielamowicz SA, Coker NJ, Jenkins HA, Igarashi M. Surgical dimensions of the facial recess in adults and children. Arch Otolaryngol Head Neck Surg. 1988;114(5):534–7. doi: 10.1001/archotol.1988.01860170064020 3355691

[19] Ansó J, Dür C, Apelt M, Venail F, Scheidegger O, Seidel K. Prospective validation of facial nerve monitoring to prevent nerve damage during robotic drilling. Frontiers in Surgery. 2019;6. 10.3389/fsurg.2019.00058 PMC6781655 31632981

[20] Ansó J, Stahl C, Gerber N, Williamson T, Gavaghan K, Rösler KM, et al. Feasibility of using EMG for early detection of the facial nerve during robotic direct cochlear access. Otol Neurotol. 2014;35(3):545–54. doi: 10.1097/MAO.0000000000000187 24492132

[21] Ansó J, Scheidegger O, Wimmer W, Gavaghan K, Gerber N, Schneider D. Neuromonitoring during robotic cochlear implantation: initial clinical experience. Annals of Biomedical Engineering. 2018;46(10):1568–81. 30051248 10.1007/s10439-018-2094-7

[22] Fosch-Villaronga E, Khanna P, Drukarch H, Custers BHM. A human in the loop in surgery automation. Nat Mach Intell. 2021;3(5):368–9. doi: 10.1038/s42256-021-00349-4

[23] Fosch-Villaronga E, Khanna P, Drukarch H, Custers B. The Role of Humans in Surgery Automation. Int J of Soc Robotics. 2022;15(3):563–80. doi: 10.1007/s12369-022-00875-0

[24] Seibel VAA, Lavinsky L, De Oliveira JAP. Morphometric study of the external and middle ear anatomy in sheep: a possible model for ear experiments. Clin Anat. 2006;19(6):503–9. doi: 10.1002/ca.20218 16287111

[25] Abari J, Al Saadi M, Van de Heyning P, Topsakal V. Defining the ideal trajectory into the inner ear in image-guided cochlear implant surgery. Sci Rep. 2024;14(1):28426. doi: 10.1038/s41598-024-79722-3 39557978 PMC11573997

[26] Feldmann A, Anso J, Bell B, Williamson T, Gavaghan K, Gerber N, et al. Temperature Prediction Model for Bone Drilling Based on Density Distribution and In Vivo Experiments for Minimally Invasive Robotic Cochlear Implantation. Ann Biomed Eng. 2016;44(5):1576–86. doi: 10.1007/s10439-015-1450-0 26358479

[27] Labadie RF, Balachandran R, Noble JH, Blachon GS, Mitchell JE, Reda FA, et al. Minimally invasive image-guided cochlear implantation surgery: first report of clinical implementation. Laryngoscope. 2014;124(8):1915–22. doi: 10.1002/lary.24520 24272427 PMC4453761

[28] Feldmann A, Wandel J, Zysset P. Reducing temperature elevation of robotic bone drilling. Med Eng Phys. 2016;38(12):1495–504. doi: 10.1016/j.medengphy.2016.10.001 27789226

